# Clinical Remarks upon Surgical Cases in the Buffalo General Hospital—Amputation of Leg—Trephining for Epilepsy—Extirpation of Melanoid Tumor

**Published:** 1865-03

**Authors:** J. F. Miner


ART. III.—*Clinical Remarks upon Surgical Cases in the Buffalo General Hospital—Amputation of Leg—Trephining for Epilepsy—Extirpation of Melanoid Tumor.* BY J. F. MINER, M. D.

*Gentlemen:*—Our first patient this morning has suffered from railroad accident, the wheels of an engine and tender having a few hours since passed over the foot, crushing it in a frightful manner; the integuments however are not so much lacerated as would have been expected, but the bones, muscles, vessels, etc. are a complete mash, and amputation is our only way of dressing. Hemorrhage has been so great, and shock so violent, that there is reason to fear he will never rally. The clinical points of greatest interest, in connection with the operation are, first, the point at which amputation shall be made, and second, the time most proper to make it; passing over of course all questions as to its necessity, since upon that point there can be no doubt. If rail cars pass over the foot, leg, or arm, while unprotected from its force, amputation is so generally a necessity that there need be little hesitation in considering what course is best to pursue; they are already amputated, as far as the circulation is concerned; and we have only to make resection in healthy tissue above the injury. The error into which you will more likely fall in your first operations after railroad accidents will be attempting to save too much.—You must remember that the death of muscle will extend several inches above the place of injury, and that the safety of your patient depends upon your making your section in tissues which are capable of life—which are healthy and uninjured.

Upon the second point of interest—the time for operating—there are a variety of opinions among surgeons which has given rise to an endless amount of discussion; which it would be improper for me now to even refer to. When called after such injuries, and you find your patient not suffering from what is called *shock* in too great a degree, if he has warmth and consciousness, and comfortable circulation, that is, distinct and somewhat forcible pulsation of the radial artery, my advice would be to proceed at once and make any necessary operation. If he is cold and pulse very feeble, and is suffering badly from what, for want of a better name, we call *shock*, then we should wait a little and stimulate and warm, and if possible animate our patient until he appears in condition of sufficient life to warrant the expectation that he will at least bear the operation we propose to make. This question of primary and secondary shock, we have no time or inclination to discuss. We have briefly indicated our rules of practice, and believe that seeing one such case as this is worth more to you—will guide you better and teach you more than any words, what course it is best to pursue, and the *time* to pursue it.

The *second case* we present this morning is a remarkable one, and we shall only attempt to give you the briefest possible account of the history, progress and present condition of this unfortunate young man, whose whole life has been rendered not only miserable, but absolutely intolerable. He is 32 years old. About twenty years ago he was accidently struck by a heavy sledge hammer, causing depression and fracture of the skull. As was to have been expected, it did not kill him, and he partially recovered from its effects. No attempt was made to raise the depressed fragments, at the time, which it appears to us now, must have been very poor surgery indeed. That it would have been proper then, there can be no doubt but that it is so now, admits of much more question. He is, however, suffering from epilepsy—a spasmodic disease about which more has been written, and less known, than any other. His paroxysms, or fits, are exceedingly frequent and violent. He is maniacal after they pass off; sometimes very violently so. Above all, he has become imbecile from their effects, and life is not only of no value to him, but is absolutely unendurable in his present condition.

We propose to trephine and raise the depressed skull, with the hope that it will at least induce some change—any change must be improvement, for he cannot be made worse. If it should relieve the irritation and pressure upon the brain, some good might follow; if it should produce inflammation and terminate in death, the operation would be a success, a great and unconditional gain.

These fragments which I present for your more careful inspection, will be observed to have projections or tubercles, which have grown from the line of fracture of the internal table, and have laid upon and even projected down through the duramater into the brain, and in connection with the pressure, have produced the effects which have been stated. The disturbance produced by removal of such a roughened mass must be very great; indeed, it now seems that in all human probability, this disturbance must produce fatal effects. If we could have foreknown the condition of the skull, and the projections which have grown from its inner surface, we might have declined the operation; but in his condition any procedure which offered the slightest prospect of relief, though attended by vast risks, was justifiable.

I have also to present you a case of rare disease—a melanotic tumor growing from the obital cavity. The eye was removed about one year since, and the appearance it presented in its commencement, and my views of the case as given before the Buffalo Medical Association, I desire to read to you, partly on account of the description and partly from personal vanity, since my early diagnosis was almost prophetic, and, unfortunately for the patient, correct:

"Dr. Miner introduced a patient with Melanosis of the eye-ball, "and gave the following history: Mr. L., aged 49 years; ob- "served imperfect vision in the right eye about one year since, "which gradually increased until in a few months he had distinct "cataract in that eye. Soon after the opacity of the lens became "visible, there also appeared a dark spot on the inner side of the "globe a few lines posterior to the junction of the cornea with the "sclerotica. This at first attracted very little attention, and

"appeared very much like a small varix of vessels in the con-"junctival membrane. July 7th, operation was made by depres-"sion of the lens, which produced no inflammatory disturbance of "the eye, and for a time promised favorable results. August 3d, "the dark spot in the conjunctiva had grown perceptibly, and effort "was made to remove it. It was found to extend into sclerotic "coat, and to be beyond the reach of removal. The lens and cap-"sule have since become involved, and now it will be observed "that the iris is changed, thickened and blackened. The mem-"branes of the globe and the contents of the eye are all involved "in melanotic disease.

"This patient had been examined by quite a number of distin-"guished surgeons while the disease was in its incipient stage; "and the patient informed that it was a very rare case, and that "they were unable to determine its character. Some of the oldest "and most experienced surgeons in the State told him that they "had never seen similar disease, and did not know what it was. "It appears a black degeneration of the tissues of the eye, rapidly "extending its borders, producing no pain or inconvenience other "than loss of sight; and opacity of the lens had previously pro-"duced total blindness, so that the melanosis cannot fairly be said "to have caused loss of vision. If it is not melanotic disease, the "question still remains; what is it? At first it might have been "regarded as staphyloma or hernia of the choroid, or pigmentary "tunic of the eye; but its rapid extension to the lens and discolor-"ation of the contents of the globe prove that it is not of such "nature, and leave no doubt as to the true character of the disease.

"It has been presented before the Society because it is a rare "form of malignant degeneration, and supposed to be of interest "to those who have never observed it, or have never seen it in its "its earlier and formative stage. When it shall become a pro-"truding blackened mass, it will lose its interest to the surgeon, "and remain only an incurable and fatal disease. The only "rational advice which can be given in the case as it now presents "itself is, extirpation of the globe of the eye. This advice will be "given this patient as soon as the disease is so fully established "as to leave no grounds for doubt, and in his opinion that period "had already arrived. It cannot, however, be urged with perfect

"assurance, that even this will overcome the disease. There is "not (as is most common) melanotic disease in other parts of the "body, so far as is known; if there is, it is of internal organs, and "gives no symptoms indicating it. Its removal may be attended "by favorable results; there are sufficient grounds of expectation "in this case, at least, to justify the measure. It is thought to be "less liable to return and less rapid in its march than encephaloid, "yet it is something of the same character, and leads to the same "results."

As I have said, the eye was removed, and you observe that the disease has re-appeared. I remove the diseased mass again, with the hope of retarding its growth, yet there are doubts that it will be productive of any favorable results whatever. That it will return again there is no doubt, and that the final termination will be delayed by a single day, is not certain.

